# Co-inhibition of PGF and VEGFA enhances the effectiveness of immunotherapy in bladder cancer

**DOI:** 10.7150/ijms.100957

**Published:** 2024-10-28

**Authors:** Xianzhi Yang, Haoxiang Zheng, Jianxu Huang, Yujun Liu, Yingrui Li, Bingwen Zhang, Chu Sun, Yuqing Li, Jean Paul Thiery, Song Wu

**Affiliations:** 1 Institute of Urology, The Third Affiliated Hospital of Shenzhen University (Luohu Hospital Group), Shenzhen 518000, China.; 2 Department of Urology, South China Hospital of Shenzhen University, Shenzhen 518116, China.; 3 Shantou University Medical College, Shantou University, Shantou, China.; 4 Medical School, Anhui University of Science and Technology, Huainan 232001, China.; 5 Guangzhou Laboratory, Guangzhou International BioIsland, Guangzhou 510005, China.; 6 BioSyngen Pte Ltd, Taiseng Exchange, 5 Tai Seng Avenue, 536671, Singapore.

**Keywords:** anti-angiogenesis, anti-PD1, PGF, bladder cancer

## Abstract

**Background:** Anti-angiogenic inhibitors and immune checkpoint blockade combination therapy offers a novel approach to circumvent the challenges associated with limited responsiveness to checkpoint inhibitors in bladder cancer. However, the effective strategies for inhibiting angiogenesis in bladder cancer need further elucidation.

**Objective:** This work aims to identify key targets for the effective inhibition of angiogenesis in bladder cancer and to explore the potential benefits of combining anti-angiogenic therapies with immune checkpoint blockade strategies in the treatment of this disease.

**Methods:** Cell-cell interaction analysis was performed using bladder cancer single-cell transcriptome datasets downloaded from the Gene Expression Omnibus (GEO) database to determine the regulatory network driving angiogenesis in bladder cancer. The bladder cancer cell line MBT2 was orthotopically transplanted into mice to investigate the impact of pro-angiogenic molecules on angiogenesis and tumor growth, and to evaluate the synergistic therapeutic potential of a combination therapy targeting angiogenesis and Programmed Cell Death Protein 1 (PD-1). Proliferation and tube formation assays with Human Umbilical Vein Endothelial Cells (HUVECs) were used to explore the regulatory functions of pro-angiogenic molecules in angiogenesis.

**Results:** Placental growth factor (PGF) is a pro-angiogenic factor in bladder cancer, in addition to vascular endothelial growth factor A (VEGFA). Suppression of PGF reduced the tumor size and angiogenesis in bladder cancer. The expression level of vascular endothelial growth factor receptor 1 (VEGFR1) is higher than that of vascular endothelial growth factor receptor2 (VEGFR2) in the endothelial cells of bladder cancer. The pro-angiogenic activity of PGF is dependent on the expression level of VEGFR1 in endothelial cells. The combined inhibition of PGF and VEGFA exerts a synergistic effect on suppressing tumor growth and angiogenesis. The concurrent inhibition of PGF and VEGFA stands out as the only intervention capable of significantly enhancing the infiltration of CD8^+^ cytotoxic T cells within the bladder cancer microenvironment. In the bladder cancer mouse model, the introduction of an anti- programmed cell death protein 1 (PD-1) therapeutic regimen combined with the targeted inhibition of PGF and VEGFA, led to a significantly elevated survival rate compared to the outcome observed with anti-PD-1 monotherapy.

**Conclusion:** PGF is a pro-angiogenic molecule in bladder cancer that requires significant expression levels of VEGFR1 in endothelial cells. Notably, the concurrent inhibition of PGF and VEGFA amplifies the therapeutic impact of anti-PD-1 treatment in bladder cancer. These findings provide further insights into the role of PGF in angiogenesis regulation and have conceptual implications for combining anti-angiogenic therapy with immune therapy in bladder cancer treatment.

## Introduction

Immune checkpoint inhibitors (ICIs) have shown survival advantages in some bladder cancer patients 1-4. Pembrolizumab has been approved by the U.S. Food and Drug administration (FDA) for non-muscle invasive bladder cancer (NMIBC) patients who exhibit refractoriness to Bacillus Calmette Guerin (BCG) 5, 6 and for patients with advanced urothelial carcinoma who are ineligible for cisplatin-containing chemotherapy 5, 7. However, this therapy has a limitation due to its low response rates, with only about 25% of the tumors demonstrating responsiveness 8-10. A common strategy to overcome this limitation is to develop combination therapies 11. For example, the National Comprehensive Cancer Network (NCCN) guidelines have incorporated the combination of pembrolizumab with the antibody-drug conjugate enfortumab vedotin as the preferred regimen for patients with locally advanced or metastatic urothelial cancer 12.

Numerous investigations demonstrate that combining an anti-angiogenic therapy with ICIs can improve the efficiency of ICIs 13, 14. The combination of the checkpoint inhibitor atezolizumab (A) with the anti-angiogenic drug bevacizumab (B) and chemotherapy (C) resulted in improved overall survival compared to AC and BC in chemotherapy-naive patients with metastatic non-squamous non-small lung cancer (NSCLC), However, this survival advantage was not observed in the AC group, suggesting a potential synergistic effect between bevacizumab and atezolizumab 15, 16. Tumor vessels lacking a basement membrane and pericytes prevent effector T cells from accessing deeper regions of tumors. Additionally, the tumor vasculature expresses high levels of immunosuppressive molecules, including programmed cell death 1 protein ligand 1 (PD-L1), resulting in the inactivation of T cells within the vascular lumen 13, 17, 18. Consequently, targeting tumor vasculature can enhance the infiltration and activation of cytotoxic T cells 19, 20. VEGFA is the critical driver of tumor angiogenesis and is the most extensively investigated target of tumor vasculature 21. However, VEGFA exhibits higher expression in pT_1_ bladder cancers than muscle-invasive (≥pT_2_) bladder cancers 22-24. This suggests tumors more likely to exhibit areas of hypoxia (e.g., Stage pT2-4) display reduced VEGFA expression 23. This finding contradicts prevailing views, as hypoxia is always associated with more angiogenesis and higher VEGFA expression 25. The clinical trials evaluating the efficacy of VEGFA inhibitors in treating bladder cancer have also shown modest benefit 26, 27. Therefore, another pro-angiogenic mechanism may be activated in bladder cancer. A better understanding of the molecular network regulating angiogenesis in bladder cancer may pave the way for future integration of checkpoint immunotherapy with anti-angiogenic therapy in the treatment of this malignancy.

In this study, we analyzed bladder cancer single-cell transcriptome datasets from the GEO database. We found that PGF is an additional pro-angiogenic factor in bladder cancer, complementing the well-known role of VEGFA. To reconcile the seemingly contradictory functions of PGF in angiogenesis, our research unveiled that the expression level of VEGFR1 in endothelial cells is a pivotal determinant of PGF's pro-angiogenic activity. Additionally, the concurrent inhibition of PGF and VEGFA, when coupled with anti-PD-1 therapy, provides a survival benefit exceeding the beneficial effect of anti-PD-1 monotherapy. These findings highlight the potential of this combinatorial approach in overcoming the limitations of checkpoint immunotherapy in bladder cancer.

## Materials and Methods

### Single-cell transcriptome datasets analysis

The GSE135337 bladder cancer single-cell transcriptome datasets, which include NMIBC and muscle invasive bladder cancer (MIBC), were downloaded from the GEO database 28. These datasets were analyzed using the Seurat package (R package, version 5.1.1). As these datasets were already normalized in the GEO database, the identification of highly variable genes and dataset integration were carried out without normalization. Principal component analysis (PCA) was performed using the RunPCA function. Significantly enriched principal components (PCs) were used for cell clustering and uniform manifold approximation and projection (UMAP) dimensional reduction. Cell clustering was performed by the FindClusters function. Clusters were annotated based on the expression of well-established marker genes for each cell type: urothelial cells (EPCAM, KRT8, and KRT18), myeloid/macrophage (CD14, CSF1R, and AIF1), T cells (CD2, CD3D, and CD3E), fibroblasts (DCN, PDPN, and TAGLN) and endothelial cells (PECAM1, VWF, and CLDN5), as reported previously 28. These cells were clustered into 21 clusters; cells of clusters 0-10, 12, 14, 16, and 20 were defined as cells of urothelial origin (bladder cancer cells); clusters 11, 13, and 18 were defined as fibroblasts; cluster 17 as T cells, cluster 15 as myeloid/macrophage, and cluster 19 as endothelial cells (Figure S1A). To explore the interaction between endothelial cells and other cell types, the Python package CellPhoneDB (version 2.1.7) was used for ligand-receptor analysis with default parameters 29. The endothelial cell subset data was then separated and an expression analysis of VEGFR1 and VEGFR2 in these endothelial cells was performed.

The GSE163558 gastric cancer (GC) single-cell transcriptome datasets were downloaded from the GEO database 30. Three tumor samples were included in our study. These datasets were also analyzed by the Seurat package. Data normalization, identification of highly variable genes, and data scaling were performed by the SCTransform function. Datasets were integrated according to the integration workflow for SCTransform in Seurat. PCA, UMAP dimensional reduction, and cell clustering analyses were done as described for the bladder cancer datasets. Cell clusters were identified using known lineage markers, such as those for epithelial cells (EPCAM, KRT18, and CLDN4), endothelial cells (PECAM1, and VWF), proliferation (STMN1, and PCNA), T cells (CD3D, CD3E, CD2), B cells (CD79A, IGHG1, and MS4A1), for natural killer (KLRD1, GNLY, and KLRF1), and myeloid (CSF1R, CSF3R, CD68), according to published data 30. Sixteen clusters were identified; cluster 8 was defined as endothelial cells (Figure S1B). The endothelial cell subset data was then separated, and an expression analysis of VEGFR1 and VEGFR2 was performed.

The GSE149614 hepatocellular carcinoma (HCC) single-cell transcriptome datasets were downloaded from the GEO database 31. Because the datasets contain a normalized expression matrix and a cell annotation matrix, the expression profiles of endothelial cells were acquired directly from these matrices. Then, the expression levels of VEGFR1 and VEGFR2 in HCC endothelial cells were analyzed.

### Immunohistochemistry

Bladder cancer tissue microarray slides were purchased from Shanghai Outdo Biotech Company (HBlaU060CS02). The tissue microarray slides were deparaffinized in xylene and then rehydrated in a series of 100%, 90%, and 70% ethanol. Antigen retrieval was performed by incubating the tissue sections with citric acid buffer (Solarbio, C1032) under a combination of high temperature and pressure within a pressure cooker for 5 minutes. Endogenous peroxidase was inactivated by incubating the sections with 3% hydrogen peroxide for 15 minutes. Then the sections were blocked for 30 min in 10% goat serum. After blocking, the sections were incubated with an anti-PGF antibody (Proteintech, 10642-1-AP, 1:200 dilution) at 4°C overnight. The next day, the tissue sections were washed with PBS three times for 5 minutes each, then incubated with an HRP-linked secondary antibody for 1 hour at room temperature, followed by incubation with Diaminobenzidine (DAB) and counterstaining with hematoxylin.

### Immunofluorescence

The multiplex immunohistochemical kit (Panovue, 10001100020) was used for immunofluorescence labeling. The procedures for deparaffinization, hydration, and antigen retrieval were the same as those for immunohistochemistry described above. Antigen detection was carried out according to the instruction manual. Tissue samples were initially treated with a blocking solution for 30 minutes, followed by an overnight incubation with the primary antibody at 4°C. Subsequently, they were incubated with the secondary antibody for 1 hour and then stained with a chromogenic agent for 10 minutes. This process was repeated for a second round of antigen retrieval, antibody incubation, and staining. Lastly, the samples were counterstained with DAPI for nuclear visualization.

### Cell lines and culture conditions

The MBT2 (RRID: CVCL_4660) mouse bladder cancer cell line was purchased from OriCell, and the human umbilical vein endothelial cells (HUVECs, (RRID: CVCL_2959)) was purchased from Biospecies. All cell lines have been authenticated using STR profiling and confirmed without mycoplasma pollution. MBT2 cell lines were cultured in RPMI 1640 medium supplemented with 10% fetal bovine serum (FBS) and 1% penicillin/streptomycin. HUVECs cell lines were cultured in complete endothelial cell medium (elGbio, EP1001). All cell lines were maintained at 37°C under 5% CO2.

### Generation of stable cell lines

The coding sequence of VEGFR1 was cloned into the pCDH vector to generate the VEGFR1-overexpression plasmid. Pgf- and Vegfa-specific shRNA oligonucleotides were inserted into the pLKO.1 to generate Pgf- and Vegfa- knockdown plasmids. Then, these plasmids were transfected into the human embryonic kidney-293 (HEK293) cells using polyethyleneimine (PEI) to produce lentiviruses. The lentiviral supernatant was harvested at 48 hours. HUVECs was infected with a VEGFR1 lentivirus using polybrene (8μg/ml) to generate a VEGFR1-overexpressing cell line. MBT2 was infected with Pgf shRNA or/and Vegfa shRNA lentiviruses, resulting in the development of MBT2 sublines with diminished expression of Pgf, Vegfa, or both. To simultaneously knockdown Pgf and Vegfa in MBT2, the pLKO vectors for Pgf shRNA and Vegfa shRNA were designed to confer distinct antibiotic resistance. Following virus infection, positive cells were selected by culturing in media containing specific antibiotics. Blasticidin was used for VEGFR1-overexpressing cells (6μg/ml, 7days), puromycin for Pgf-knockdown cells (8μg/ml, 2days), hygromycin for Vegfa-knockdown cells (800μg/ml, 4days). The knockdown of Pgf and Vegfa, as well as the overexpression of VEGFR1, were validated by western blot analysis probing these proteins. The sequences of primes are provided in supplementary materials (Table S1,2).

### Tumor model and treatment regimens

Cells of the MBT2 sublines, suspended in DMEM devoid of FBS and antibiotics, were mixed with Matrigel (Corning, 354262) at a ratio of 100: 15, then orthotopically implanted into the bladder wall of C57BL/6 mice (2.5x10^5^ cells/mouse). The C57BL/6 mice purchased from Gempharmatech were administered intraperitoneal injections of phosphate-buffered saline (PBS) and the anti-mouse PD-1 antibody (200μg/mouse, SELLECK, A2122).

### Western blot

Western blotting was performed according to standard procedures. Cells were washed with ice-cold PBS and then lysed using 1×SDS lysis buffer. The samples were boiled, resolved in 12% Tris-glycine gels, and transferred to a polyvinylidene difluoride membrane using wet transfer. The polyvinylidene difluoride membranes were blocked with 5% milk in Tris-buffered saline with 0.02% Tween20 (TBS-T) for 1 hour. They were then probed with primary antibodies overnight at 4°C, followed by incubation with a secondary antibody conjugated to horseradish peroxidase for 1-2 hours at room temperature. The secondary antibody was diluted in 2.5% milk in TBS-T. The antibodies used for western blot were as follows: anti-PGF (1:500, Proteintech, 10642-1-AP), anti-VEGFA (1:500, Proteintech, 19003-1-AP), anti-VEGFR1 (1:1000, HuaBio, ET1605-11), Anti-phosphorylated ERK1/2 (1:500, Servicebio, GB11004), and anti-ERK1/2 (1:1000, Servicebio, GB11560). The specific proteins were detected using a ChemiDoc^TM^MP Imaging System (Biorad) after incubation with the western HRP substrate (Beyotime, P0018S).

### Proliferation assay

HUVECs cell lines were seeded into a 96-well plate (2000 cells/well) and cultured with complete endothelial cell medium. To explore the effect of PGF on endothelial cell proliferation, the complete endothelial cell medium was replaced with either a standard endothelial cell medium depleted of growth factors or a medium containing 100 ng/ml of PGF (MCE, HY-P70749). Cell numbers were determined using the CCK8 assay (Abbkine, BMU106-CN) according to the standard procedure. In brief, 10 μl CCK8 solution was added to each well. Then, the cells were incubated at 37°C for 2 hours. The absorbance was detected using the iMark^TM^ Microplate Reader (Biorad).

### Tube formation assay

To assess the effects of PGF on the tube formation ability of HUVECs cell lines, 96-well angiogenesis plates (Ibidi, 89646) were coated with 10μl Matrigel (Corning, 356231). Then, the plates were incubated at 37°C for at least 30 minutes to allow matrigel polymerization. The HUVECs cell lines cultured in distinct conditions described above for 3 days were seeded into the plates (8000 cells/well) and incubated at 37°C under 5% CO_2_. 8 hours later, these HUVECs cell lines were imaged using an Incucyte S3 system (ESSEN Bioscience).

### Bulk RNA seq and analysis

Total RNA was extracted using the TRIZOL reagent according to the standard procedure. The mRNA was purified using poly-T oligo-coupled magnetic beads, fragmented, and cDNA was synthesized and converted into a library for sequencing. Libraries were sequenced on an Illumina Novaseq platform, and 150bp paired-end reads were generated.

Raw data of fastq format was processed. In this step, clean data was obtained by removing reads containing adapter, poly-N, and low-quality reads from raw data. At the same time, Q20, Q30, and GC content of the clean data were calculated. The paired-end reads were aligned to the reference genome using Hisat2 v2.0.5. FeatureCounts v1.5.0-p3 was used to count the reads numbers mapped to each gene. Then, the FPKM of each gene was calculated based on the length of the gene and the read counts mapped to this gene. Differential expression analysis of two conditions was performed using the edgeR R package (3.22.5). Gene Ontology (GO) enrichment analysis of differentially expressed genes was performed using the DAVID website (https://david.ncifcrf.gov/tools.jsp). GO terms with p-values lower than 0.05 were considered significantly enriched. Gene Set Enrichment Analysis (GSEA) was performed using GSEA_4.3.2 software.

### Flow cytometry analysis

Mouse bladder tumors were cut into small pieces and incubated in a digestion buffer comprising 0.1% collagenase I, 0.25% collagenase IV, and 1mg/ml hyaluronidase at 37°C, 200rpm for 2 hours to obtain single-cell suspensions. The cell suspensions were incubated in red cell lysis buffer for 10 minutes to remove red blood cells and then they were filtered through a 40 μm cell strainer. The dissociated cells were stained with the following primary antibodies: anti-CD45 (Biolegend, 157606), anti-CD3 (Biolegend, 100204), anti-CD8a (Biolegend, 100708), anti-CD4 (Biolegend, 100407), and anti-CD25 (Biolegend, 101915). The cells were further permeabilized using True-Nuclear Transcription Factor Buffer set (Biolegend, 424401) and stained with anti-FOXP3 (Biolegend, 126419). Flow cytometry was performed using a BD LSRFortessa^TM^ X-20 flow cytometer (BD Biosciences), and the data were analyzed using FlowJo software version 10.

### Statistical analysis

All statistical analyses were performed using the GraphPad Prism 8.0 software. Differential expressions of FLT1 and KDR were assessed using a paired two-tailed T test. Differences in survival rates were calculated using the Log-rank (Mantel-Cox) test. Differences among multiple groups were initially assessed using one-way ANOVA, followed by comparisons between each pair of groups using Tukey's multiple comparisons test. Differences in all other categories were analyzed using an unpaired two-tailed T-test. Values were presented as mean±SD. Statistical significance was set at P<0.05.

## Results

### FLT1-PGF interaction between endothelial cells and carcinoma cells is highly expressed in both NMIBC and MIBC

**Figure 1:**
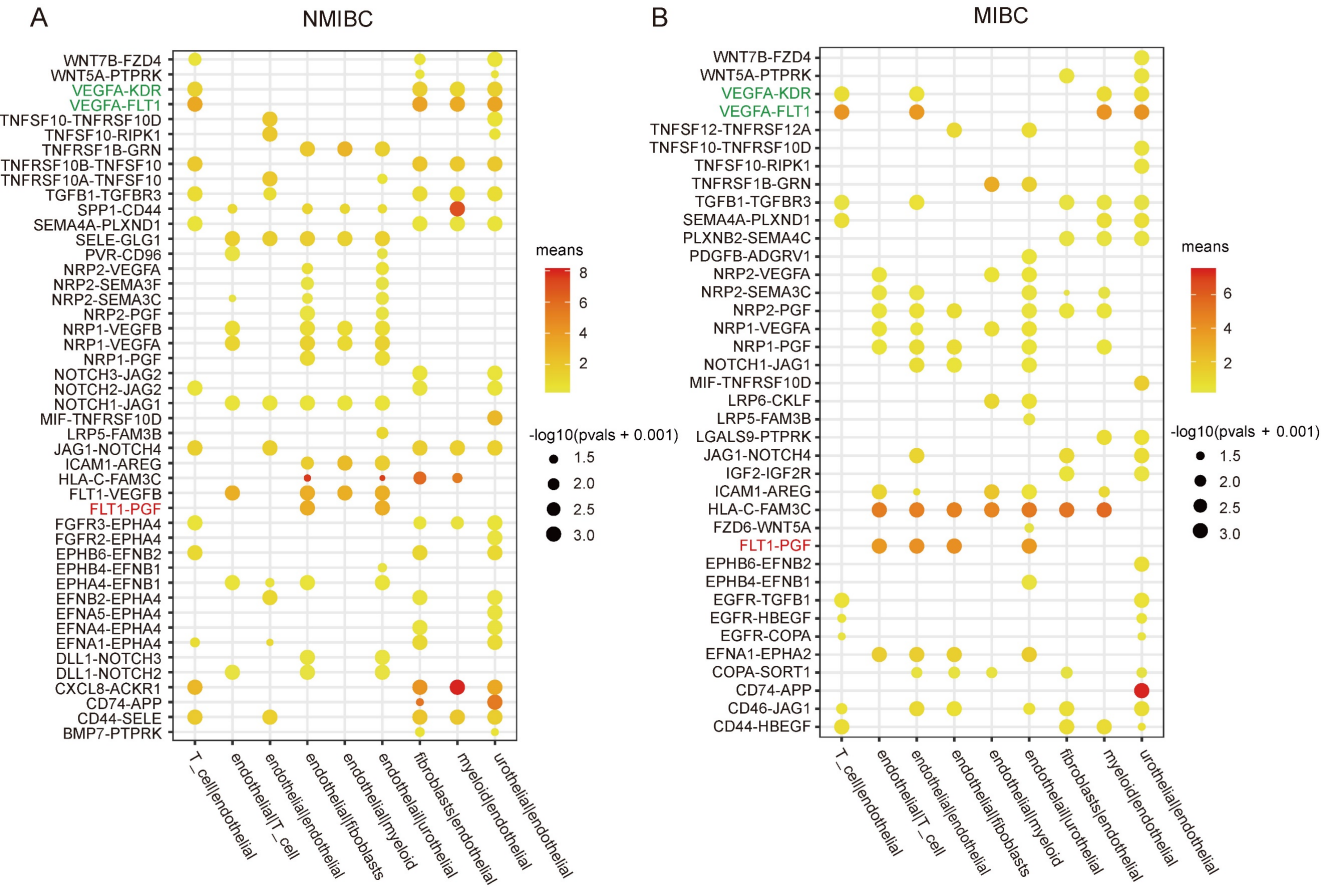
** Cell-cell interaction analysis of NMIBC and MIBC.** (A, B) The ligand-receptor interactions between endothelial cells and other cell populations are significantly enriched (p-value < 0.05) in NMIBC and MIBC. The intensity of dot colors corresponds to the expression level, while the size of the dots reflects the statistical significance (p-value).

To elucidate the angiogenic regulatory mechanisms operating in bladder cancer, we analyzed single-cell transcriptome datasets of bladder cancer obtained from the GEO database. The CellphoneDB package was used to explore the ligand-receptor interactions between endothelial cells and other cell populations, based on expression levels of ligands and receptors within distinct cell clusters. Interestingly, the pro-angiogenic ligand-receptor interactions of FLT1(VEGFR1)-PGF, FLT1(VEGFR1)-VEGFB, ACKR1-CXCL8, and FLT1(VEGFR1)-VEGFA between endothelial cells and urothelial-derived carcinoma cells were enriched and displayed higher expression levels compared to other interactions in NMIBC (Figure 1A); however, only FLT1(VEGFR1)-PGF and FLT1(VEGFR1)-VEGFA exhibited higher expression in MIBC (Figure 1B). PGF belongs to the VEGF family, which also includes VEGFA, B, C and D 32. Therefore, this finding suggests that PGF may play an important role in angiogenesis in bladder cancer, in addition to VEGFA.

VEGFA is known to bind to FLT1 (VEGFR1) and KDR (VEGFR2). However, VEGFA-FLT1 interaction surprisingly exceeded VEGFA-KDR interaction in both NMIBC and MIBC, even though KDR is recognized as the primary receptor for VEGFA-induced angiogenesis 33. As the results derived from CellphoneDB were based on the expression levels of ligands and receptors in distinct cell types, this phenomenon could be attributed to the expression levels of FLT1 and KDR in the endothelial cells of bladder cancers (Fig. 1A.B).

### PGF promotes angiogenesis in bladder cancer

**Figure 2:**
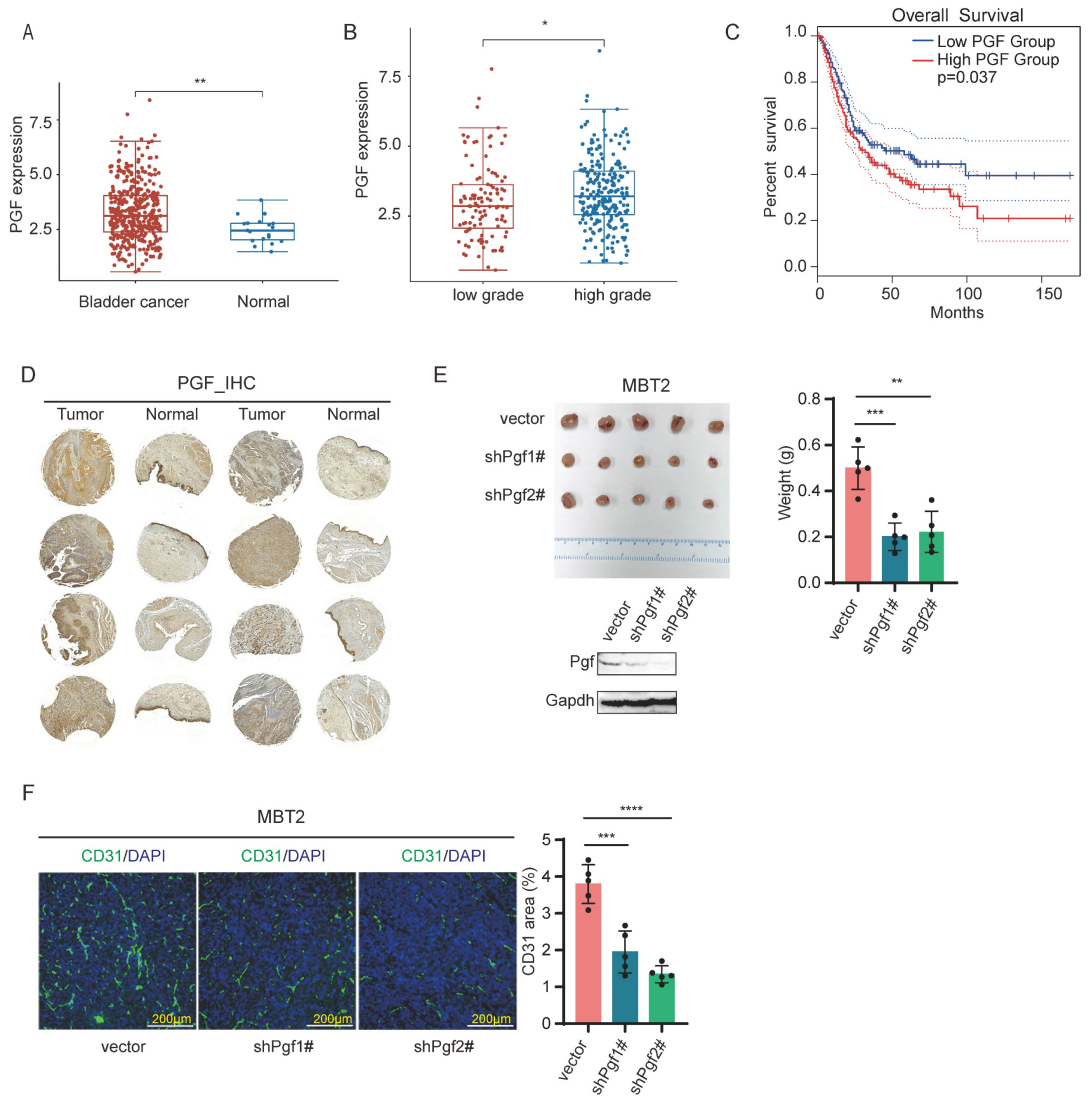
** PGF is a pro-angiogenic factor in bladder cancer.** (A) Statistical analysis of mRNA levels of PGF in bladder cancer patients using TCGA datasets (Wilcox test). (B) Comparison of mRNA levels of PGF between high-grade and low-grade bladder cancer using TCGA datasets (Wilcox test). (C) Survival analysis of bladder cancer patients stratified by PGF expression levels using data from TCGA. (D) Immunohistochemical analysis of PGF expression in a clinically representative tissue microarray of bladder cancer samples. (E) Tumor size analysis of MBT2 with targeted knockdown of Pgf. (F) Immunofluorescence analysis of the vessel density (CD31). The histogram represents quantitative statistics of the fluorescent area. * p< 0.05, ** p <0.01, *** p <0.001, **** p <0.0001.

To explore whether PGF serves as a pro-angiogenic factor in bladder cancer, we analyzed its expression levels and assessed its correlation with patient survival rate through Gene Expression Profiling Interactive Analysis 2 (GEPIA2) web platform (http://gepia2.cancer-pku.cn/#index) 34 and Assistant of Clinical Bioinformatics web platform (https://www.aclbi.com/static/index.html#/). These analyses were conducted using bladder cancer clinical datasets from The Cancer Genome Atlas Program (TCGA) database. The expression levels of PGF are elevated in tumor samples relative to normal tissue counterparts (Figure 2A), and in bladder cancer, high-grade tumors (>pT2) present higher PGF expression compared to low-grade tumors (<=pT2) (Figure 2B). A negative correlation between PGF expression levels and overall survival was observed in bladder cancer patients (Figure 2C). These findings underscore the significance of PGF in the context of bladder cancer pathology. To further validate this, immunohistochemical staining of PGF expression was conducted using a tissue microarray of clinical bladder cancer specimens. The expression levels of PGF were elevated in tumor samples when compared to matched normal bladder tissues (Figure 2D). Additionally, *in vivo* studies using a mouse model orthotopically transplanted with MBT2 demonstrated that knockdown of Pgf led to a reduction in tumor size (Figure 2E). Histological analysis of bladder tissues from these mice, using immunofluorescence labeling of CD31, revealed a decrease in vessel density following the silencing of Pgf (Figure 2F).

### The pro-angiogenic activity of PGF depends on the expression levels of VEGFR1 in endothelial cells

The role of PGF in angiogenesis remains unclear. Previous studies have shown that PGF can positively or negatively modulate angiogenesis. The overexpression of PGF has been reported to lead to the inhibition of angiogenesis and the normalization of blood vessels, which in turn suppresses tumor growth 35. However, other studies have found that PGF contributes to pathological angiogenesis with little impact on normal vasculature 36, 37. PGF exclusively activates FLT1, also known as VEGFR1. VEGFR1 is a receptor tyrosine kinase (RTK) exhibiting lower kinase activity than VEGFR2 38. It was thought to act as an angiogenic antagonist by competing with VEGFR2 for VEGFA binding 39, 40. However, VEGFA, a well-established pro-angiogenic factor, can activate both FLT1 and KDR, also known as VEGFR2. VEGFR2 is considered the predominant receptor mediating the pro-angiogenic activity of VEGFA 41. The expression level of VEGFR2 is usually higher than that of VEGFR1 in normal blood vessels 42. Surprisingly, the expression level of VEGFA-FLT1(VEGFR1) interaction between bladder cancer cells and endothelial cells is higher than that of VEGFA-KDR(VEGFR2) interaction (Figure 1). As the results derived from CellphoneDB are based on the expression profiles of ligands and receptors 29, the expression level of FLT1 in the endothelium of bladder cancer might be higher than that of KDR. The higher expression of FLT1 might contribute to the pro-angiogenic activity of PGF in bladder cancer. This could explain why PGF does not affect normal vessels or even inhibits angiogenesis in some studies but activates angiogenesis in bladder cancer.

**Figure 3:**
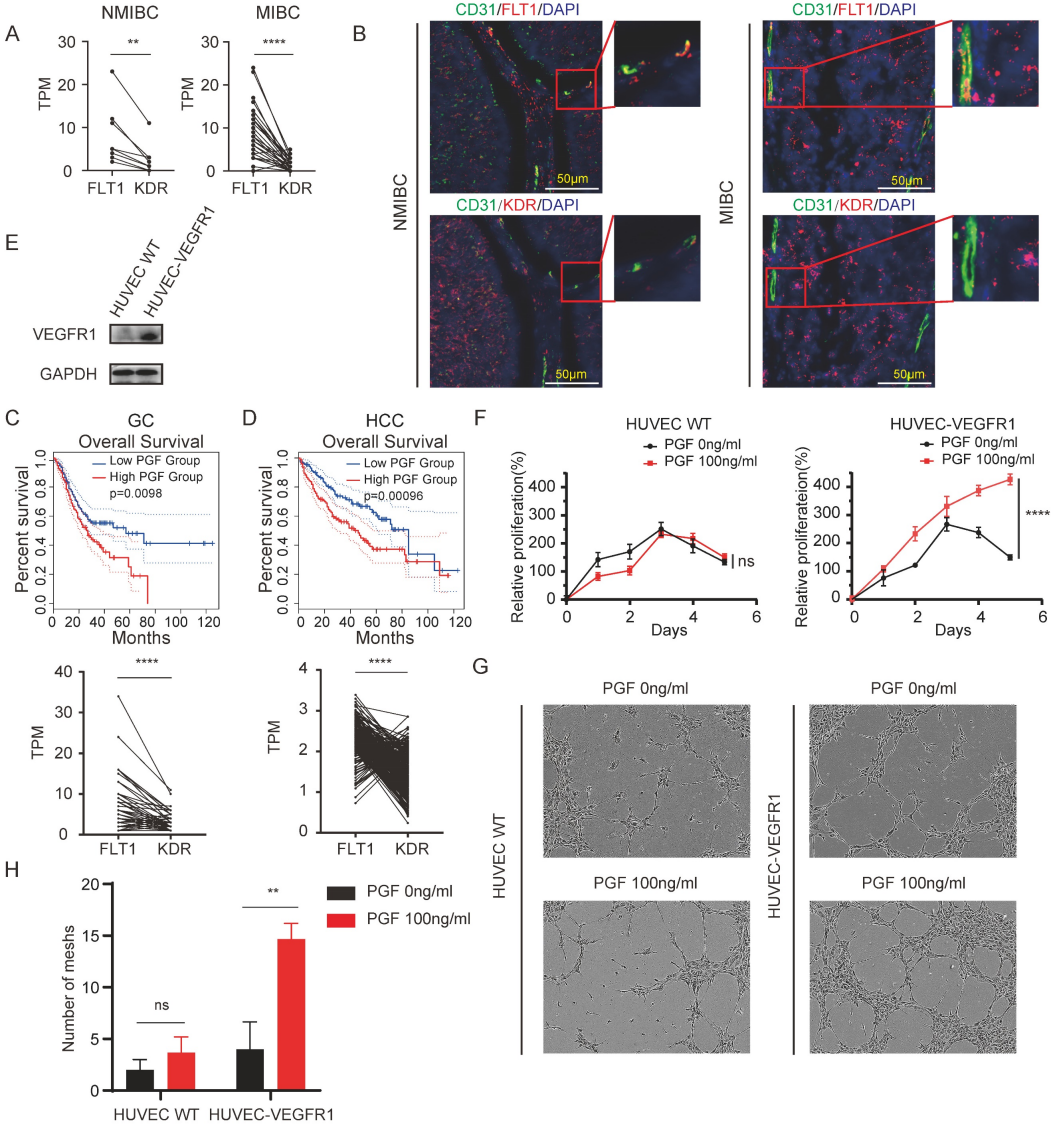
** The pro-angiogenic activity of PGF is dependent on the expression level of VEGFR1 in endothelial cells.** (A) The expression levels of FLT1 (VEGFR1) and KDR (VEGFR2) in endothelial cells of NMIBC and MIBC. (B) Immunofluorescence analysis of CD31, VEGFR1, and VGFR2. (C, D) The expression of FLT1 and KDR in GC and HCC in which high expression of PGF is associated with poor survival rate. (E) VEGFR1 overexpression validation in HUVECs. (F) Proliferation curves of VEGFR1-overexpressing HUVECs (HUVEC-VEGFR1) and wild-type HUVECs (HUVEC WT) incubated with PGF protein or not. (G, H) Images of distinct tube formation patterns and quantitative analysis using ImageJ software revealed significant differences in loop numbers across three independent replicates. ns means no significant difference. ** p < 0.01, **** p < 0.0001.

To test this hypothesis, an expression analysis of FLT1 and KDR in endothelial cells was performed using single-cell transcriptome datasets. Notably, the expression level of FLT1 was significantly higher than that of KDR in both NMIBC and MIBC (Figure 3A). This finding was further validated via immunofluorescence double staining of CD31 with FLT1 or KDR in clinical bladder cancer tissues. The results confirmed a greater degree of co-localization between CD31 and FLT1 compared to that between CD31 and KDR (Figure 3B). The high expression of FLT1 coupled with low expression of KDR in endothelial cells is also observed in gastric cancer (GC) and hepatocellular carcinoma (HCC), where the expression level of PGF inversely correlates with patient overall survival rates (Figure 3C, D). To further evaluate the role of VEGFR1, it was genetically overexpressed in HUVECs (Figure 3E). PGF did not activate proliferation and tubule formation in wild-type HUVECs, but significantly stimulated these processes in HUVECs overexpressing VEGFR1 (Figure 3F, G, H). These results support the contention that the expression level of VEGFR1 plays a crucial role in mediating the pro-angiogenic activity of PGF in bladder cancer.

### ERK1/2 are robustly activated upon PGF engagement with VEGFR1

**Figure 4:**
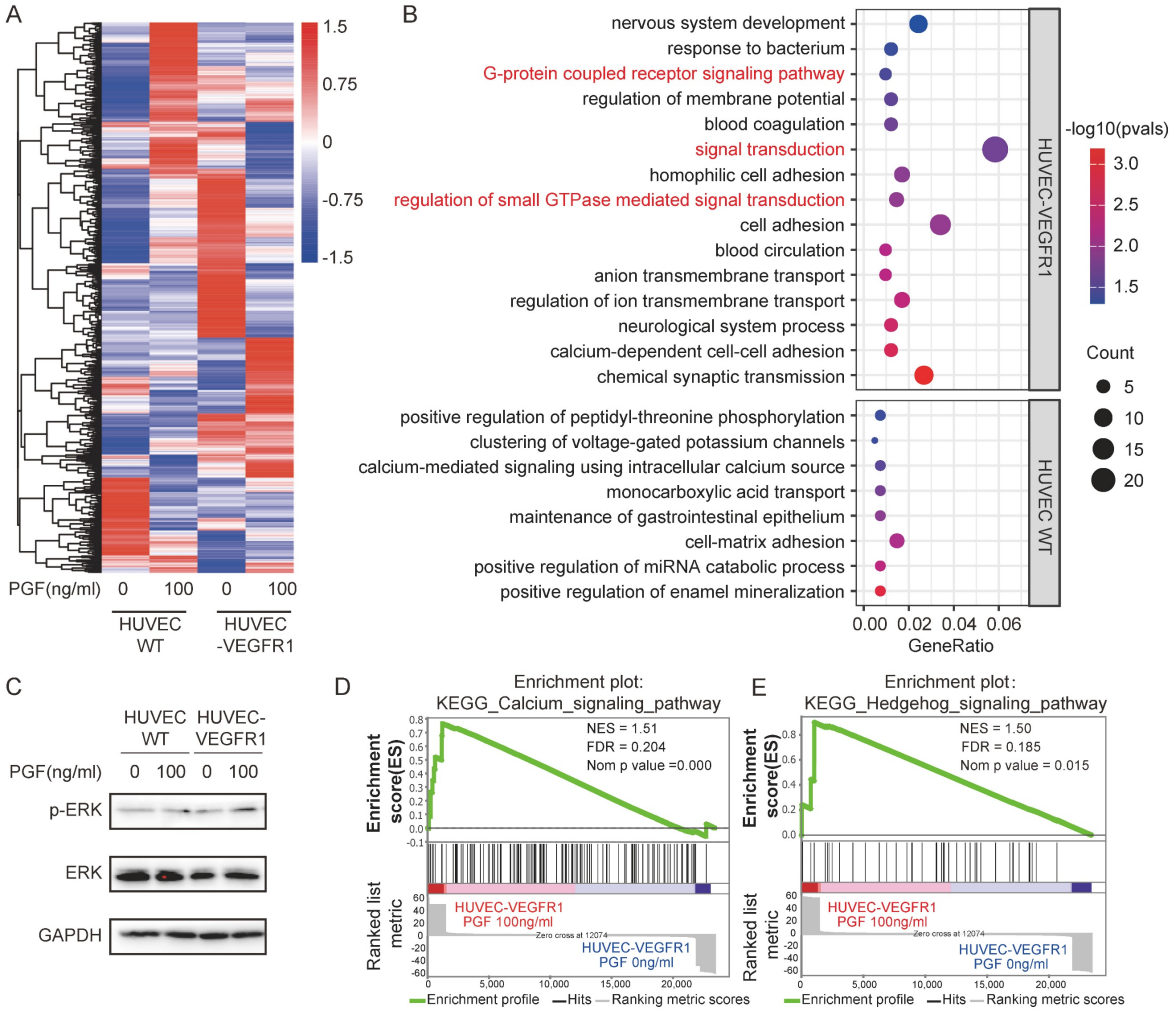
** Signal transduction pathways mediate the pro-angiogenic activity of PGF.** (A) Heatmap displaying the expression pattern of differently expressed genes in response to PGF in HUVEC WT and HUVEC-VEGFR1. (B) GO analysis focusing on differently expressed genes in response to PGF in HUVEC-WT and HUVEC-VEGFR1. (C) Western blot probing phosphorylated ERK1/2. (D.E) GSEA plot for “KEGG calcium signaling pathway” and “KEGG hedgehog signaling pathway” in HUVEC-VEGR1 cells.

To delineate the impact of PGF stimulation on endothelial cells, an RNA-seq analysis was carried out to map the comprehensive changes in their transcriptomic landscape. The differential gene expression profiles following PGF stimulation diverged markedly between VEGFR1-overexpressing HUVECs and its wild-type counterpart (Figure 4A). According to a Gene Ontology (GO) enrichment analysis focusing on the differently expressed genes in response to PGF stimulation in VEGFR1-overexpressing HUVECs and wild-type HUVECs, a notable enrichment of several signaling transduction pathways was exclusively found in the VEGFR1-overexpressing HUVECs. These pathways included the regulation of small GTPase-mediated signal transduction, signal transduction, and the G-protein coupled receptor signaling pathway, all of which are related to vascular endothelial growth factor receptor (VEGFR) activation (Figure 4B) 43-45. Therefore, the high expression of VEGFR1 is essential for efficient PGF signaling, consistent with previous findings.

ERK1/2 activation is an essential downstream event in the angiogenic cascade orchestrated by VEGFR2 46, 47. ERK1/2 has also been identified as a downstream target of VEGFR1 signaling in monocytes and human retinal endothelial cells 48, 49. We found that in VEGFR1-overexpressing HUVECs, PGF treatment led to the activation of ERK1/2, whereas this effect was absent in wild-type HUVECs (Figure 4C). This observation implies that ERK1/2 may act as a key downstream effector of VEGFR1 in mediating the pro-angiogenic effects of PGF, bridging receptor activation to the subsequent pro-angiogenic signaling. Additionally, GSEA analysis revealed that the calcium and hedgehog pathways were enriched in VEGFR1-overexpressing HUVECs treated with PGF (Figure 4D, E). Both pathways are associated with the process of angiogenesis 50-52.

### Co-inhibition of PGF and VEGFA enhances the effectiveness of anti-PD-1 antibodies

**Figure 5:**
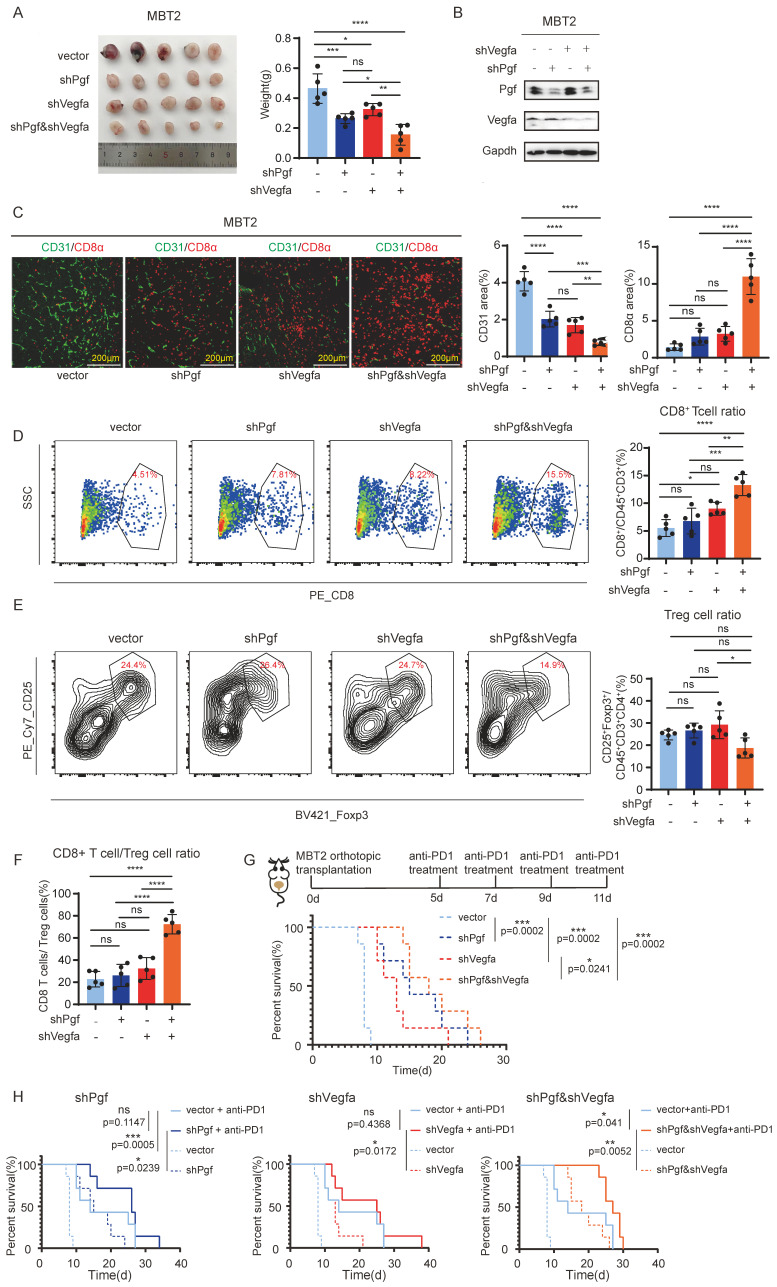
** The combined inhibition of PGF and VEGFA significantly augments the survival outcome when administered alongside anti-PD1 antibody therapy.** (A) Tumor size analysis of MBT2 with targeted knockdown of Pgf and/or Vegfa orthotopically implanted into the mouse bladder. (B) Western blot probing the PGF and VEGFA. (C) Immunofluorescence analysis of vessel density (CD31) and infiltration of CD8^+^ T cell (CD8). Histograms represent quantitative analysis of fluorescent areas. (D, E) Flow cytometry analysis of the CD8^+^ T cell and Treg within the microenvironment of MBT2 xenografts. The histogram depicts the quantitative analysis of positive cells. (F) Calculation of the CD8^+^ T cell to Treg cell ratio based on the above flow cytometry data. (G, H) Survival analysis evaluating the therapeutic efficacy of anti-PD1 antibody treatment in mice bearing MBT2 tumors with targeted knockdown of Pgf and/or Vegfa, and the treatment schedule. * p<0.05, ** p<0.01, *** p <0.001, **** p<0.0001, ns means no significant difference.

According to the results described above, PGF is shown to be another pro-angiogenic factor in bladder cancer. This discovery offers insight into the downregulation of VEGFA observed during disease progression, suggesting that PGF may compensate for the reduced VEGFA activity. Therefore, co-inhibition of PGF and VEGFA could potentially achieve synergistic anti-angiogenic effects. To validate this hypothesis, both Vegfa and Pgf were knocked down in MBT2 (Figure 5B). Co-inhibition of Vegfa and Pgf resulted in the most pronounced reduction in tumor size and the lowest density of CD31- positive blood vessels (Figure 5A, C).

As reported in previous studies, anti-angiogenic therapies increased the infiltration of CD8^+^ cytotoxic T cells into tumors, potentiating the effectiveness of immunotherapy against cancer 19. In line with these findings, we investigated the impact of concurrently targeting Vegfa and Pgf on the infiltration of immune cells within the tumor microenvironment. We found that only the co-inhibition of Vegfa and Pgf markedly increased the infiltration of CD8^+^ cytotoxic T cells (Figure 5C, D). Even though the infiltration of regulatory T cells (Tregs) was not significantly affected by the co-inhibition of Vegfa and Pgf (Figure 5E), the ratio of CD8^+^ T cell to Treg cell within the tumor was significantly elevated when Vegfa and Pgf were concurrently knocked down (Fig.5F). The individual blockade of Vegfa resulted in minimal change in CD8^+^ T cell infiltration, but did not affect the CD8^+^ T cell to Treg cell ratio within the tumor (Figure 5 D, F). Therefore, only concurrent inhibition of both Vegfa and Pgf induces significant alterations in the composition of immune cells within the tumor microenvironment. Thus, the combinatorial effect of anti-PD-1 antibody treatment and pro-angiogenic molecule silencing was evaluated. Our findings indicate that the dual inhibition of Vegfa and Pgf, as well as their individual blockades, significantly enhanced the survival rate of mice with bladder cancer. Notably, the co-inhibition of Vegfa and Pgf yielded a superior survival benefit when compared to the single inhibition of Vegfa alone. However, there was no significant difference in survival rates between the single inhibition of Pgf and the combined inhibition of Vegfa and Pgf (p-value is not shown) (Figure 5G). Only the co-inhibition of Vegfa and Pgf significantly enhances the survival benefit associated with anti-PD1 antibody therapy in the context of bladder cancer management (Figure 5H).

## Discussion

Checkpoint immunotherapy has contributed to the treatment of bladder cancer, but its effectiveness still needs to be further improved. In recent years, there has been a resurgence of interest in anti-angiogenic therapy, recognizing its potential to augment the effectiveness of checkpoint immunotherapy. Combining immune checkpoint inhibitors with anti-angiogenic therapies is likely an approach to enhance the efficacy of checkpoint immunotherapy in bladder cancer treatment. To date, clinical trials using angiogenic inhibitors in the treatment of bladder cancer have not been successful. To our knowledge, almost all anti-angiogenic therapies target the VEGFA-VEGFR2 signaling pathway. However, VEGFA expression is reduced during the progression of bladder cancer, altering the use of anti-angiogenic agents that target VEGFA/VEGFR2 signaling. These findings highlight the need for further investigations into the mechanism of angiogenic regulation in bladder cancer.

We found that PGF promotes angiogenesis in bladder cancer in addition to VEGFA, and the combined inhibition of PGF and VEGFA yields synergistic effects in tumor growth inhibition and anti-tumor immunity. Anti-angiogenic therapies have been underutilized in the broader spectrum of cancer treatment due to their historically limited effectiveness. Our findings suggest that the ineffectiveness of current anti-angiogenic therapies, which mainly focus on inhibiting the VEGFA/VEGFR2 signaling axis, might result from an incomplete angiogenesis blockade. This is because the impact of VEGFA/VEGFR2 pathway inhibitors can be counteracted by activating alternative pro-angiogenic pathways.

The expression level of VEGR1 is higher than that of VEGFR2 in bladder cancer, contributing to the pro-angiogenic activity of PGF in this disease. The mechanism underlying the expression profiles of VEGFR1 and VEGFR2 is unknown. We have demonstrated that this expression pattern is not unique to bladder cancer. It is hypothesized that these distinctive expression profiles could indicate the unique characteristics of tumor blood endothelial cells, which are crucial to the formation of immature and permeable tumor vasculature. Furthermore, the hypoxic conditions prevalent within the tumor microenvironment, which are known to foster angiogenesis, may be responsible for the high VEGFR1 and low VEGFR2 expression levels. It is also plausible that these expression patterns are organ-specific, reflecting the unique physiological demands of different tissues. Exploring these hypotheses is crucial for understanding whether similar synergistic effects from the co-inhibition of PGF and VEGFA occur in other types of cancer, beyond bladder cancer, and warrants further investigation.

Unlike VEGFA, there is a lack of clinically available anti-PGF antibodies for *in vivo* inhibition. The effectiveness of antibody-mediated PGF inhibition relative to its genetic ablation remains unknown. Notably, PB101, a glycosylated soluble decoy receptor fusion protein, was developed to antagonize both VEGFA and PGF. PB101 demonstrated robust decoy activity against these key angiogenic factors 53. In the tumor model, PB101 not only efficiently inhibited the tumor angiogenesis and progression 53, 54, but also enhanced antitumor immunity 55, consistent with our findings. Future works should focus on developing drugs specifically inhibiting PGF signaling or, ideally, PGF and VEGFA, and on exploring the synergistic effects of combining these drugs with immunotherapy for more effective clinical treatments of bladder cancer.

## Supplementary Material

Supplementary figure and tables.

## Abbreviations

FDA: the U.S. Food and drug administration
GC: gastric cancer
HCC: hepatocellular carcinoma
HUVECs: human umbilical vein endothelial cells
ICIs: immune checkpoint inhibitors
MIBC: muscle invasive bladder cancer
NCCN: the National Comprehensive Cancer Network
NMIBC: non-muscle invasive bladder cancer
PD-1: programmed cell death protein 1
PD-L1: programmed cell death 1 protein ligand 1
PGF: placental growth factor
RTK: receptor tyrosine kinase
Tregs: regulatory T cells
VEGFA: vascular endothelial growth factor A
VEGFR1: vascular endothelial growth factor receptor1
VEGFR2: vascular endothelial growth factor receptor2
