# Supplementary material for: Co-inhibition of PGF and VEGFA enhances the effectiveness of immunotherapy in bladder cancer
Source: Int J Med Sci. 2024 Oct 28;21(15):2870–82. doi: 10.7150/ijms.100957 (PMC11610333; doi:10.7150/ijms.100957)
Supplement: Supplementary file 1 — Supplementary figure and tables. [file ijmsv21p2870s1.pdf]

Supplemental figure1:

Figure.S1:(A) Cell clustering and annotation of bladder cancer single-cell transcriptome dataset. (B) Cell clustering and annotation of gastric cancer single-cell transcriptome dataset.

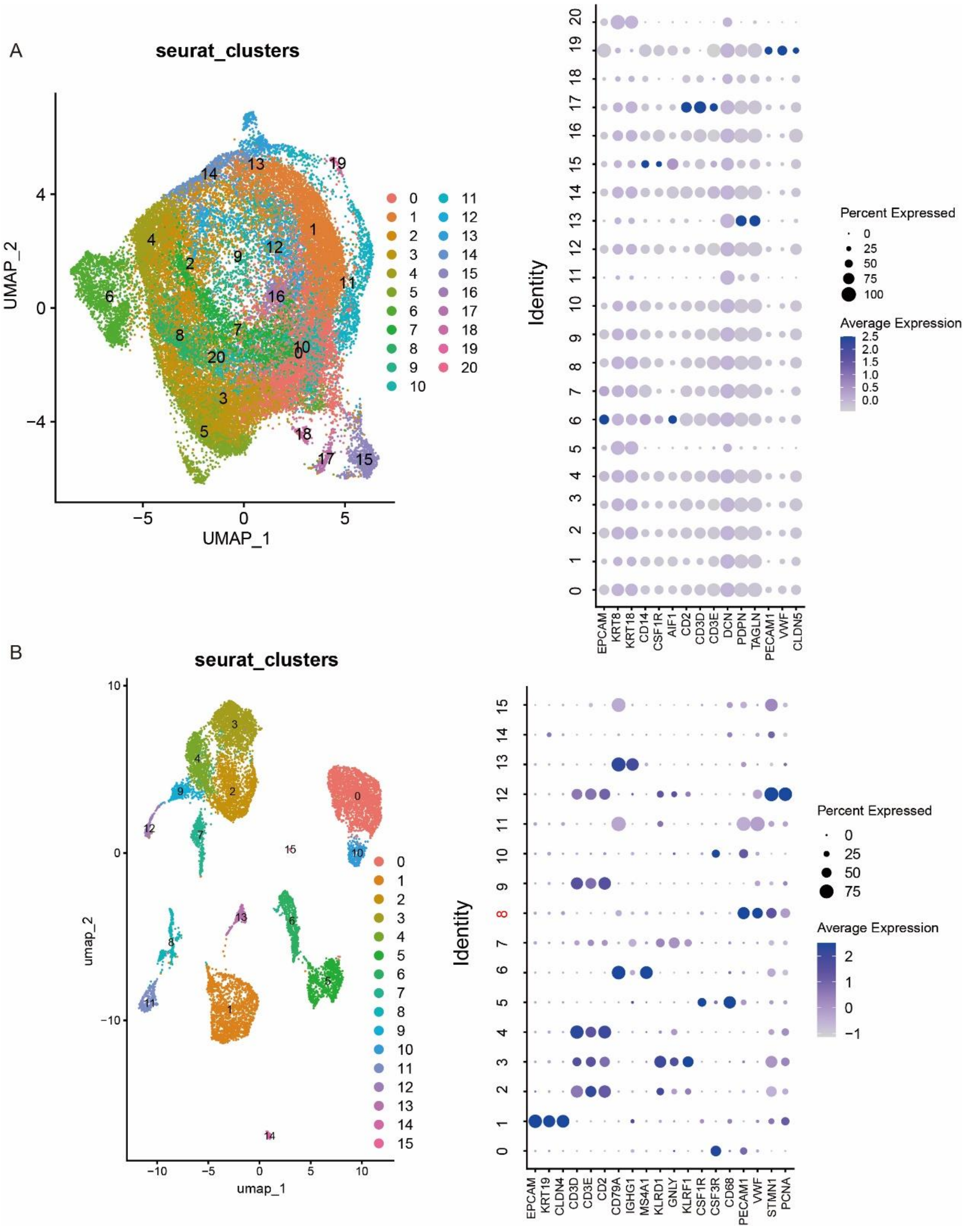

Supplemental table1:

Table S1: The primers used to amplify VEGFR1.

Name	Sequence
homo-ECOR1-VEGFR1-F	GATTCTAGAGCTAGCGAATTCATGGTCAGCTACTGGGACACC
homo-XHO1-VEGFR1-R	ACGTCGTAGGGGTAACTCGAGTAGATGGGTGGGGTGGAGTACAG

Supplemental table2:

Table S2: The shRNA sequence of this paper.

Name	Sequence
mouse-Pgf-sh1-f	CCGGCTCTGTGAATGCAGACCTATTCTCGAGAATAGGTCTGCATTCACAGAGTTTTTG
mouse-Pgf-sh1-r	AATTCAAAAACTCTGTGAATGCAGACCTATTCTCGAGAATAGGTCTGCATTCACAGAG
mouse-Pgf-sh2-f	CCGGTCCACATTTCTATGTGGAGATCTCGAGATCTCCACATAGAAATGTGGATTTTTG
mouse-Pgf-sh2-r	AATTCAAAAATCCACATTTCTATGTGGAGATCTCGAGATCTCCACATAGAAATGTGGA
mouse-vegfa-sh1-f	CCGGGCAGACCAAAGAAAGACAGAACTCGAGTTCTGTCTTTCTTTGGTCTGCTTTTTG
mouse-vegfa-sh1-r	AATTCAAAAAGCAGACCAAAGAAAGACAGAACTCGAGTTCTGTCTTTCTTTGGTCTGC
